# Retrosplenial cortex in spatial memory: focus on immediate early genes mapping

**DOI:** 10.1186/s13041-021-00880-w

**Published:** 2021-12-04

**Authors:** Edyta Balcerek, Urszula Włodkowska, Rafał Czajkowski

**Affiliations:** Laboratory of Spatial Memory, Nencki Institute of Experimental Biology, Polish Academy of Sciences, Pasteura 3, 02-093 Warsaw, Poland

**Keywords:** Immediate early genes, *c-Fos*, *Arc*, *Homer*, Retrosplenial cortex, Spatial memory

## Abstract

The ability to form, retrieve and update autobiographical memories is one of the most fascinating features of human behavior. Spatial memory, the ability to remember the layout of the external environment and to navigate within its boundaries, is closely related to the autobiographical memory domain. It is served by an overlapping brain circuit, centered around the hippocampus (HPC) where the cognitive map index is stored. Apart from the hippocampus, several cortical structures participate in this process. Their relative contribution is a subject of intense research in both humans and animal models. One of the most widely studied regions is the retrosplenial cortex (RSC), an area in the parietal lobe densely interconnected with the hippocampal formation. Several methodological approaches have been established over decades in order to investigate the cortical aspects of memory. One of the most successful techniques is based on the analysis of brain expression patterns of the immediate early genes (IEGs). The common feature of this diverse group of genes is fast upregulation of their mRNA translation upon physiologically relevant stimulus. In the central nervous system they are rapidly triggered by neuronal activity and plasticity during learning. There is a widely accepted consensus that their expression level corresponds to the engagement of individual neurons in the formation of memory trace. Imaging of the IEGs might therefore provide a picture of an emerging memory engram. In this review we present the overview of IEG mapping studies of retrosplenial cortex in rodent models. We begin with classical techniques, immunohistochemical detection of protein and fluorescent in situ hybridization of mRNA. We then proceed to advanced methods where fluorescent genetically encoded IEG reporters are chronically followed in vivo during memory formation. We end with a combination of genetic IEG labelling and optogenetic approach, where the activity of the entire engram is manipulated. We finally present a hypothesis that attempts to unify our current state of knowledge about the function of RSC.

## Introduction

The year 2021 marks the 50th anniversary of the discovery of physiological coordinates of the spatial map in the brain—place neurons tuned by the rat’s position within the experimental compartment [1]. Apart from place cells in the hippocampus, grid and border neurons in the medial entorhinal cortex, the system also includes head direction neurons [2]. Head directionality is represented within several structures, including mammillary bodies in the hypothalamus [3] and the anterior thalamic nuclei [4]. It has also been recorded in the cortical regions, most notably in the presubiculum [5] and the retrosplenial cortex [6].

Since the late nineteenth century, when Brodmann first identified anatomically the retrosplenial cortex, substantial experimental evidence supporting its function has accumulated. Although the precise function of RSC is still not yet fully determined, results from both human and animal studies clearly point to a prominent role in spatial cognition. The neuroanatomical, behavioral and electrophysiological experiments in animal models, as well as the functional and neuropathological studies in human subjects have been the subject of a number of excellent reviews [7–12]. After a brief recapitulation of this body of knowledge, our review will focus on the results of a very specific, yet powerful approach to RSC studies in animal models—the mapping of immediate early genes.

**Fig. 1:**
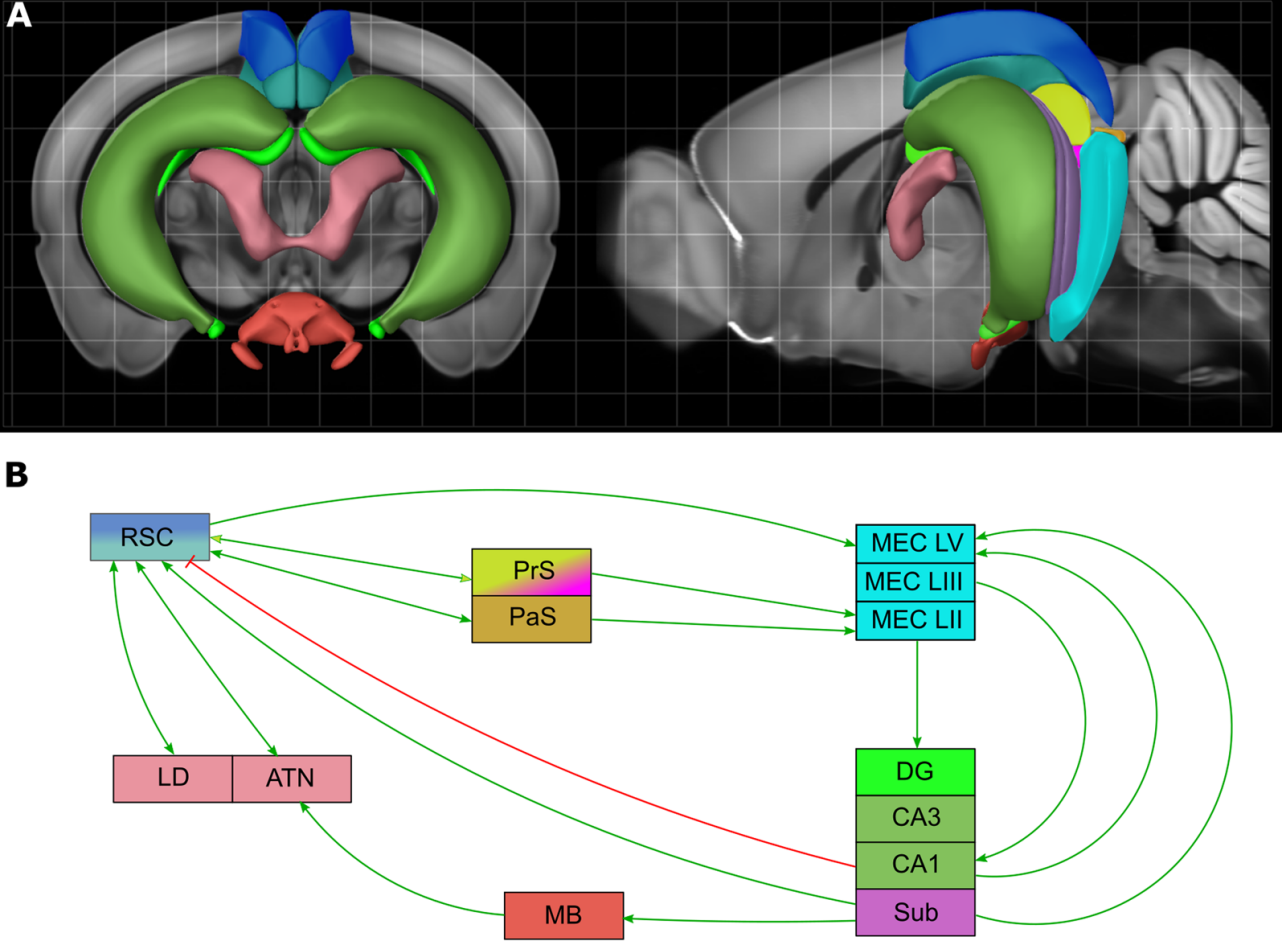
Position of retrosplenial cortex within the spatial memory circuit. **A** Simplified drawing of relative localization of anatomical structures within the rodent brain (composition of 3D renderings and 2D images from Mouse Common Coordinate Framework. Image credit: Allen Institute). See colors in B for legend. **B** Simplified diagram of connectivity. *RSC* retrosplenial cortex, *DG* dentate gyrus, *CA1, CA3* hippocampal (*corni ammonu*) subdivisions, *Sub* subiculum, *PrS* presubiculum (postsubiculum was incorporated as dorsal part of presubiculum), *PaS* parasubiculum, *MEC* medial entorhinal cortex, *ATN* anterior thalamic nuclei, *LD* laterodorsal thalamic nuclei, *MB* mammillary bodies

RSC is the cortical region that Brodmann delineated as separate areas 29 and 30, which in humans form a complex with areas 23 and 31 of posterior cingulate cortex. Studies of distribution of cortical projections, first with anterogradely transported radioactively labeled amino acids, and then with diverse retrograde tracers revealed that RSC is strongly connectected with the key brain regions contributing to the memory system (Fig. 1). One of fundamental findings was that the parahippocampal region is heavily interconnected with RSC. The dorsal part of the subiculum (Sub) gives rise to ipsilateral connections to RSC [13, 14] and return projections to the pre- and parasubiculum are known [15]. Granular retrosplenial cortex (RSG) receives direct inputs from the dorsal hippocampus, primarily from subiculum and secondarily from CA1 [16, 17], both glutamatergic [18] and GABAergic [19, 20]. Direct efferents of dorsal subiculum terminate densely in layer II and superficial layer III across granular area, contrasting with much lighter terminations in agranular retrosplenial cortex [21]. These anatomically proven pathways have also been tested functionally [18]. RSC is positioned at the interface between sensory cortical regions and structures that compose the parahippocampal-hippocampal memory network. Importantly, the connections between RSC and those structures are both afferent and efferent, suggesting that RSC not only directs incoming sensory information to the hippocampus, but may also serve as a crucial site of information storage. Medial entorhinal cortex neurons receive inputs from the retrosplenial area [22, 23]. Evident reciprocal connections from anterior and lateral thalamic nuclei (ATN and LD) to RSC have been identified on the basis of cytoarchitecture labeling [24, 25] or lesion studies [26]. Agranular retrosplenial cortex (RSA), which is often referred to as the dysgranular part of retrosplenial cortex, receives direct sensory inputs from visual areas 17 and 18 [27]. Studies of limbic circuitry showed also that retrosplenial cortex projects bilaterally to the medial region of the mammillary nuclei [28]. Prominent reciprocal connections between RSC and prefrontal cortex [29, 30] and between RSC and posterior secondary motor cortex have been also described [31].

The most striking evidence about functional engagement of candidate brain regions in spatial memory often comes from the human lesion cases. RSC lesioned patients demonstrated deficits of personal autobiographical memories having intact general intellectual function [32]. Evidence that RSC could act as an interface between memory retrieval and visuospatial processes was also described [33]. Topographical disorientation and navigation deficits following damage of retrosplenial cortex were identified, showing evidence for strong right retrosplenial lateralization [12]. Patients with small focal hemorrhages localized in the right retrosplenial area showed deficits in orientation and great difficulty with spatial positional relationships between two locations within a familiar area [34]. Cases of memory impairment in patients with damage of left RSC have also been noticed [35, 36].

Functional neuroimaging studies of human brain activity have identified cortical regions, including retrosplenial cortex, parahippocampal cortex and posterior and medial parietal cortices, that respond more strongly during virtual or imagined navigations compared to non-navigational control tasks [37–39]. Engagement of RSC in retrieval of spatial information was confirmed by study of Epstein and colleagues [40] who showed stronger response of RSC when subjects had to judge the locations and orientation than when they retrieve simple familiarity. Moreover, the response of RSC was stronger during location judgments than during orientation. Identification of familiar scenes showed stronger RSC activity than when viewing unfamiliar spatial surroundings. Study of taxi drivers who moved around central London in a virtual environment shows entire RSC activation during both route planning and spontaneous decisions to change their route during navigation [41].

Anatomical lesions in animal models initiated a chapter in spatial memory studies showing the importance of the hippocampus for proper navigation [42]. Among a variety of tasks, water maze performance, which depends on allocentric cues, clearly shows effects of particular structure modifications. Further studies revealed that damage of entorhinal cortex also impairs acquisition in water maze, rising the idea that this structure mediates cortical sensory information transfer to HPC [43]. On the other hand there were reports showing that entorhinal cortex damage does not change the ability to solve spatial tasks sensitive to hippocampal cell loss [44]. The fact that connections between RSC and main memory structures like thalamus and entorhinal cortex exist could explain the remaining source of cortical sensory information that supports hippocampal processing. A number of RSC lesion studies of rats and nonhuman primates showed impaired spatial navigation [45–50]. There have been some reports showing no evidence of straightforward impairments of spatial navigation after RSC damage [51, 52]. Those lesion interventions have very rarely removed the entire region, the most caudal section was generally intact [47, 53, 54]. Targeted lesions of the most caudal part of the posterior cingulate cortex in nonhuman primates impaired ability to retrieve information that has been previously acquired [55]. Some retrosplenial subregions appear to have greater engagement in spatial processing than others. For example, spatial deficits occurred when lesions included a region of area 29b, but not 29a. Area 29b receives direct input from the visual association cortex (area 18b) and this connection may be a key component of RSC role in landmark navigation [56].

The development of electrophysiological recordings in animal models allowed for further exploration of RSC involvement in neural circuitry underlying spatial navigation. The hippocampus, entorhinal and retrosplenial cortices are the main brain structures that have been implicated in building an internal map of the environment. Location, distance and direction in the environment are represented in the brain by place cells, grid cells and head direction cells respectively [57]. The electrophysiological recordings of rodent RSC have identified neurons that encode allocentric representation of location. This small population of about 10% cells, called head direction cells (HD), fire when animals maintain certain heading within an environment regardless of position and become silent when the head points in all other directions [5, 6, 58]. The HD signal is dependent on environmental landmarks and the strength of this dependency is relative to perception of the stability of the particular cue [59]. Some HD neurons of dysgranular retrosplenial cortex are supposed to mediate between existing local visual landmarks and the global HD signal. Such neurons having opposing HD tuning curves could use existing directional representation to compute head direction allowing for encounter of new landmarks and removal of unstable ones [60]. Taking into consideration strong connections of MEC and RSC, it is possible to speculate that RSC head-direction cells may also control grid field orientation [57]. Subpopulation of neurons mostly from superficial layers of RSC express a sparse, continuous and spatially localized representation of the full length of a linear environment which is highly similar in its properties to the population of hippocampal CA1 place cells. RSC place neurons fired in reproducible sequences during movement but not during stillness [61]. Place neurons in RSC were active independently of tactile stimuli but depended critically on the intact hippocampus [62–64]. RSC neurons appear to mediate relationships among the multiple forms of spatial information. RSC space encoding is highly sensitive to route position relative to environment boundaries often referred to as the allocentric frame of reference and simultaneously dependent on specific left and right turning actions [45]. RSC neurons have been identified to represent fragmentation of complex route via the same shape of spatially periodic activation patterns. On the other hand, a larger population of RSC neurons provides a novel metric for distance between all route locations having single cycle periodicity over the full route of plus-shaped track [65]. Importance of looking at the RSC by wider neuronal network reference was highlighted by cross frequency activity modulation and interactions between RSC and hippocampus during REM sleep [66, 67].

The lesion studies, as well as the electrophysiological recordings in the rodent model, provided a diverse but sometimes contradictory and inconsistent picture of the RSC function in memory formation. The main drawback of the lesion approach was its highly intrusive nature. Irreversible damage to entire structures exposed unwanted effects, and the requirement for post-lesion recovery could lead to compensatory mechanisms. Pharmacological inactivations solved the problem only partially, the non-specific nature of these interventions targeting the entire structure remained problematic. Electrophysiological recordings suffered from relatively low sampling rate and from the lack of long term stability of the recorded ensembles. The problem of the dynamic and lengthy process of memory formation called for novel approaches that would allow for drawing a more global picture.

## Immediate early genes

Development of new molecular biology tools in the early 1980s led to the discovery of protooncogenes and to understanding their function as transcription factors instrumental for initiation of long term cellular response. It was proposed that this type of phenomenon might occur in neurons and provide the mechanism for learning related plasticity [68]. Several researchers—most notably Kaczmarek [69], Morgan, Curran [70] and Kandel [71]—independently suggested that exploiting this potential connection between gene expression and memory formation might lead to establishing new methods of investigating learning-related phenomena, with resolution increased from brain regions to the level of circuits and possibly individual cells [72]. The presence of nuclear protein c-FOS had been at that time already observed in several of cell activation phenomena [68]. That, coupled with the discovery of presence of c-FOS [73] and then its inducibility in the nervous system [74] made it a good candidate for a possible marker of neuronal plasticity. The *c-Fos* gene (in rodents usually simply referred to as *Fos* or FOS in the case of the protein product) is often classified as an immediate early gene. Expression of the genes that belong to this group is activated rapidly after stimulation while in quiescent animals it remains very low. Major contributions to understanding the relationship between gene function and neural plasticity have been made due to study of protein synthesis inhibition in various time windows after stimulation [75].

The behavioral effect of inhibition of cerebral protein synthesis has been studied since the 60 s, when the influence of intracerebral injection of the antibiotic puromycin on learning in mice was first described [76]. In the decades following the first study, hundreds of reports on pharmacology of memory have been published, leading to consensus that pre training blocking of de novo protein synthesis does not impair short term task retention, only influencing long term memory. Drugs administered post training—which also means independently of task acquisition—also influence long term retention, the shorter the training—treatment interval, the more pronounced the effect [77]. The discussed timing is in line with Kaczmarek’s hypothesis of replenishment of resources rapidly depleted in response to bursts of activity preceding learning as an explanation for memory related gene expression [75].

**Fig. 2:**
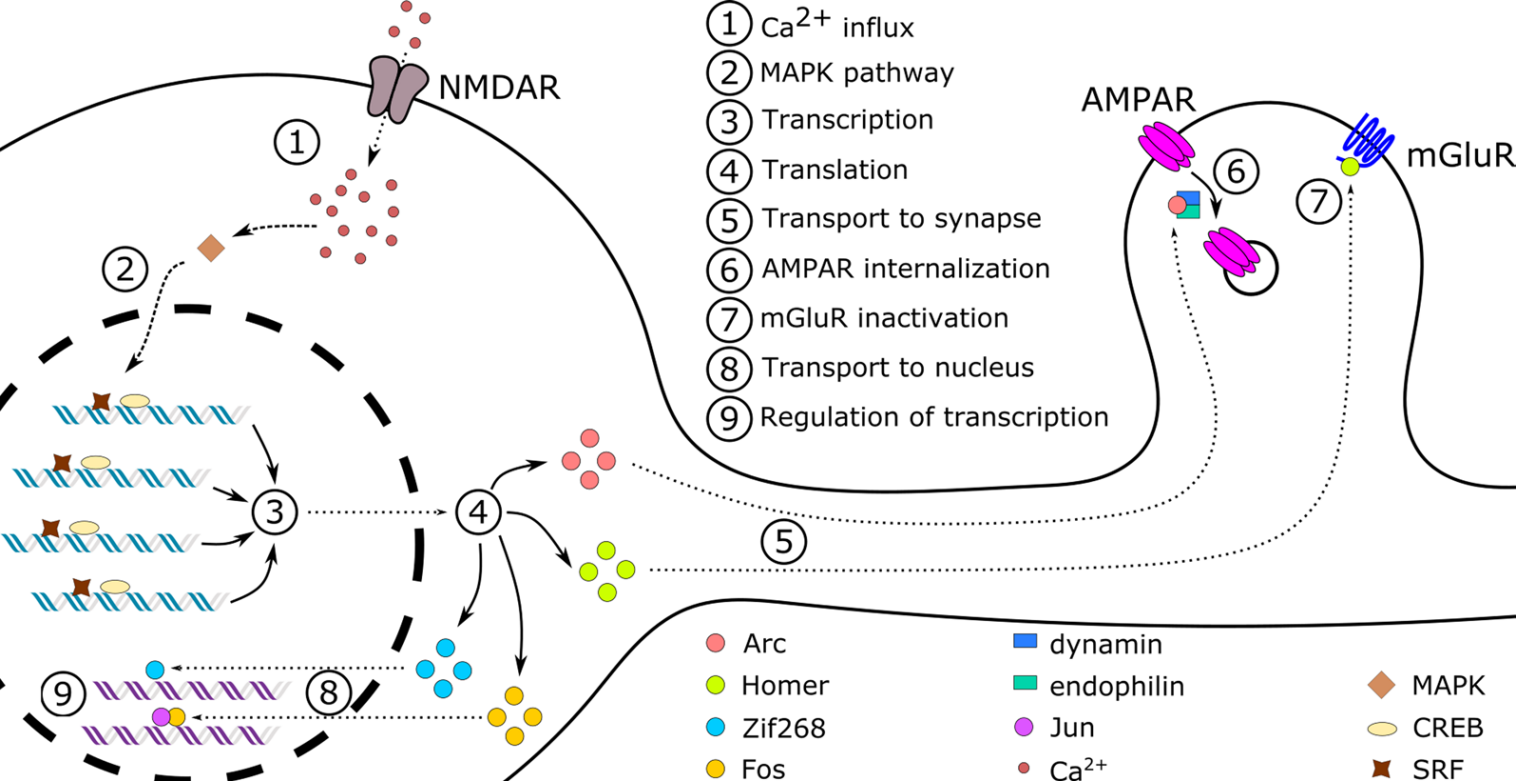
Simplified diagram of IEG signalling pathways. NMDAR-mediated Ca^2+^ influx leads to activation of signalling pathways transporting the signal to the nucleus. MAPK pathway-activated protein kinases phosphorylate CREB and ELK-1 transcription factors that bind regulatory elements of the immediate early genes. *Arc* and *Homer* mRNA is transported to the synapse, where it is translated. ARC downregulates AMPAR by interacting with endophilin and dynamin and enhancing the receptor endocytosis. Homer 1a selectively binds group 1 metabotropic receptors, thus, it competes with constitutively expressed CC-Homers and disassembles the signaling complex, inactivating mGluR. The c-FOS and ZIF268 proteins are transported back into the nucleus, after translation in the cytoplasm. Inside the nucleus, downstream gene transcription is regulated by ZIF268 and AP-1 complex, which is a dimer of c-FOS and c-JUN

IEGs encode an array of proteins which differ in their function (Fig. 2). Among the first ones to be examined in the field of learning and memory are the activity-induced regulatory transcription factors (RTF) subset. Those proteins enter the nucleus, where they regulate transcription of other genes. Notable examples of activity-induced RTFs are *Fos*, *Jun* and *Krox* gene families [78, 79]. Members of FOS and JUN families dimerize with each other to form parts of activator protein-1 (AP-1) complex. The AP-1 is one of the most thoroughly studied transcription factors in the central nervous system [80] and its involvement in the plastic phenomena of the brain is well documented [80–82].

*Krox* family became of interest for the learning and memory field due to discovery of *Krox-24* gene [83], also known as *Zif268* [84], zinc-finger containing transcription factor expressed in the brain both constitutively and after seizures [85]. It has been proven that ZIF268 protein is induced in the hippocampus by the same activity patterns that induce long term potentiation (LTP) [86]. Moreover, both ZIF268 presence and LTP are dependent on NMDA (N-methyl-d-aspartate) receptor activity.

Those early studies on IEGs present in the brain after seizures led to development of novel IEG identification methods by providing an understanding of stimulus types and intensity thresholding that allows for inducing a more diverse set of genes belonging to this group [87].

Wide ranging search for neuronal IEGs by cloning and identification of transcription factors induced in the brain after maximal electroconvulsive shock (MECS) led to division of IEGs into two groups. Apart from RTF neuron-specific IEGs, there exist so-called effector IEGs. They are characterized by the ability to directly modify the cellular function. In the late 90 s it was proposed that effector IEGs might influence synaptic plasticity through regulation of structure, signal transduction ability or spatial localization of critical receptors [87]. A representative of this group of IEGs, *Arc* (activity-regulated cytoskeleton associated protein, also known as *Arg3.1*) has been of particular interest in the learning and memory research field. It was discovered in the mid 90 s independently by Kuhl and Worley groups [88, 89]. *Arc* expression in hippocampal neurons is observed to be tightly coupled with stimulation both in the form of MECS as well as behavioral induction in hippocampus-dependent tasks [90]. Following an inducing stimulus, *Arc* mRNA is quickly distributed to distal parts of the dendritic tree, which allows for its synapse-specific function (Fig. 2). Further investigation led to discovery that aforementioned types of stimuli lead to *Arc* being expressed exclusively in neurons—which sets this IEG apart from other well studied ones. After induction, ARC protein is found solely in αCaMKII expressing principal neurons of the hippocampus, striatum and neocortex [91]. Interestingly, *Arc* does not show homology to any other gene nor belongs to a family of genes—which suggests that it evolved relatively late and has a highly specific role [78]. It has been recently proposed that ARC emerged as a repurposed retrotransposon GAG protein, acquiring a novel function within the CNS [92].

*Homer* (Homer protein homolog 1) is another effector IEG with a unique mechanism directly targeting synapses. It is induced in the visual cortex during development, and experimentally in the hippocampus in association with long term potentiation, as well as in striatum in response to chemical stimulation with drugs altering dopamine signaling. It interacts with the C terminus of mGluR5 (Metabotropic glutamate receptor 5) and early in vitro studies on rat hippocampal extracts (naive and after MECS) suggested that neural activation might modify the affinity of this interaction [87, 93, 94]. The original *Homer* sequence (now commonly referred to as *Homer 1a*) has been used in search for other members of a possible gene family. This led to the discovery of a group of genes called *CC-Homers* that are constitutively expressed in the brain and compete with *H1a* in forming signalling complexes [95] (Fig. 2).

## Classical IEG studies

**Fig. 3:**
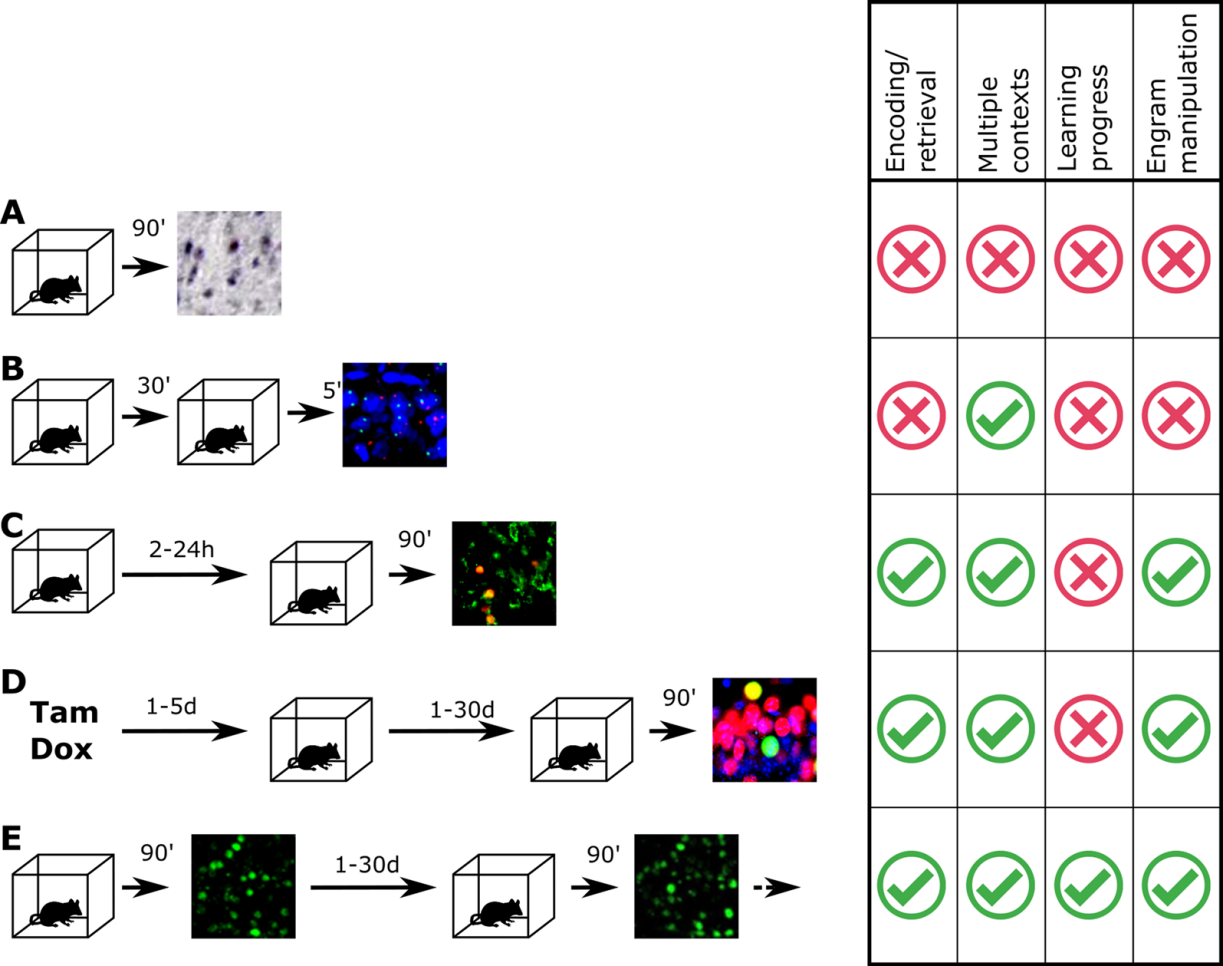
The development of experimental approaches towards detection of IEG engram and their applications in the study of spatial memory. **A** Immunohistochemistry (or immuofluorescence) detection of FOS protein after behavioral session. **B** Compartment analysis of temporal activity by fluorescent in-situ hybridization (catFISH) of *Arc* and *Homer* mRNA (courtesy of W. Karwicka and A. Hamed). **C** Co-detection of *Fos* fluorescent reporter transgene and FOS protein (courtesy of K. Andraka and E. Knapska). **D** Co-detection of pharmacologically controlled *Fos* reporter transgene and FOS protein (courtesy of J. Wilmot and B. Wiltgen). **E** Chronic in vivo imaging of FOS fluorescent IEG reporter

The expression of immediate early genes (IEGs), particularly *c-Fos, Arc* and *Zif268* has been used as a marker of neural activity in a vast number of spatial memory studies. For over two decades, most of the experiments relied on immunohistochemical detection in the fixed tissue, harvested 90–120 min after the behavioral session, when the IEG protein level peaks. It only allowed for measuring one time point per experimental group (Fig. 3A). This approach was further improved by the use of stable fluorescent markers driven by an IEG promoter in conjunction with native IEG detection [96] (Fig. 3C). A similar system was additionally expressed under the tetracycline-dependent system, allowing for more precise temporal control and prolonged separation between sessions [97] (Fig. 3D). Another solution was based on precise analysis of the timing of transcription activation and mRNA processing (Fig. 3B). It allowed for development of a powerful technique called cellular compartment analysis of temporal activity by fluorescent in-situ hybridization (catFISH). One variant of this method took advantage of differences in time interval between stimulus and peak levels of different IEG mRNAs (*Arc* and *Homer*). Another one used solely *Arc*—the first time point, associated with activity immediately before tissue collection, was represented by intranuclear transcription foci, the second one—30 min prior—by cytoplasmic *Arc* mRNA [78, 90]. It makes it possible to identify and quantify neuronal populations activated by two distinct experiences separated by minutes. In all three cases, neuronal ensembles tagged in two separate timepoints could be compared for each animal.

### Allocentric navigation

Given the somewhat peripheral position of RSC within the navigation circuit (Fig. 1), the initial *Fos* mapping studies including this structure were often a part of a broader anatomical picture. Similarly, the behavioral paradigms were not specifically designed to address RSC involvement. The very first extensive report of this kind showed changes in *Fos* expression induced by progressively more demanding variants of radial arm maze task. There was an increased number of FOS-positive nuclei in RSC of rats performing the standard radial arm maze compared to the group running up and down a single arm in the same maze. Performing the same task in a novel room did not result in any further FOS protein increases in this region. It led to the conclusion that the retrosplenial cortex is a part of the working spatial memory circuit, possibly involved in tracking the animals’ position in relation to the environment rather than processing novel spatial stimuli [98]. Retrosplenial cortex would be involved in updating the position of the animal by monitoring its trajectory in relation to a start location.

Another study using a similar working memory radial-arm maze task showed that there is evident difference between activity of FOS protein in granular and dysgranular subregions of the retrosplenial cortex. Radial arm maze task consistently increased *Fos* activity in granular retrosplenial cortex irrespective of whether the spatial memory task was in the light or dark. The dysgranular retrosplenial cortex was engaged only when the task was carried out with light on. That may indicate selective involvement of dysgranular retrosplenial cortex when distal visual cues control performance and that granular retrosplenial cortex contributes to spatial learning and navigation based on both internal and external cues [99].

Following the paper by Vann [98], a number of studies used the *Fos* mapping to address the interconnectivity of RSC with other structures known for involvement in spatial memory and navigation. A report tracking changes in *Fos* activity upon bilateral anterior thalamic lesions shows that retrosplenial activity depends on the integrity of the anterior thalamic nuclei. FOS protein levels were measured after rats had been placed in a novel room in preselected arms of a radial maze. Such exposure was sufficient to increase *Fos* activity of RSC in control groups, whereas the group with anterior thalamic lesion showed a significant decrease in FOS counts in RSC [100]. Moreover, unilateral anterior thalamic lesions were sufficient to decrease *Fos* active cells in ipsilateral HPC and RSC. Those changes seemed to be linked with areas involved in spatial memory processing and required for proper task performance [101]. Irrespective of inactivation method (excitotoxin or radiofrequency current) there was a striking loss of FOS-positive cells in the granular RSC of rats performing radial-arm maze in a novel room. Dysgranular retrosplenial cortex did not initially show this decrease of FOS-positive cells. The vast majority of affected neurons was found in the superficial laminae weeks after surgery. The deeper layers of granular RSC remained unchanged. The observed hypoactivity in the superfcial layers of granular RSC after anterior thalamic lesions remains persistent and did not appear to be task specific. Similar change was found in rats taken directly from the home cage. Moreover, data collected 9–10 months postsurgery show chronic effects of those lesions. Rats with anterior thalamic lesions and exposure to activity box for 30 min showed decrease of both FOS and ZIF268 throughout the RSC, with dysgranular part also affected [102]. Lesions of mammillothalamic tract (MTT), a white matter bundle, which sends unidirectional projections from the mammillary bodies to the anterior thalamic nuclei impaired T-maze alternation performance of rats. Beyond the behavioral effect, MTT lesions decreased levels of ZIF268 and a more general metabolic marker, cytochrome oxidase, in RSC. The lesions decreased levels of both activity markers in the superficial and deep layers of the RSC in both its granular and dysgranular subregions. Despite the behavior impairments of T-maze alternation no significant changes were observed in the hippocampus [103]. Disruption of memory upon MTT damage was also reflected in the pattern of *Fos* hypoactivity in RSC of rats performing forced runs in a radial-arm maze in a novel room. The greatest FOS decrease was observed in both the superficial and in the deep layers for all retrosplenial subregions, except for the very deep layers of granular RSC [104]. Unlike the previous study, this one reported decrease in FOS measured activity in HPC after MTT lesions.

Hippocampal formation damage itself produces permanent dysfunctions in retrosplenial cortex activity of rats performing several experimental paradigms (different lesion method, different rat strain, different behavioural task). Striking losses of *Fos* and *Zif268* activity were observed in both superficial and deep laminae of all retrosplenial subregions. Superficial layers (II and upper III) displayed most dramatic decreases in FOS-positive cells, there was an additional band of cells in layer V of the granular retrosplenial cortex that appeared to be largely deprived of FOS-positive cells. Despite the decrease of FOS and ZIF268 protein levels, there was no evidence of any changes in cellular size, shape or appearance in RSC [105]. The same study shows that entorhinal cortex lesions had no effect on retrosplenial IEGs levels. Another study of spatial memory in water maze task used fluorescence in situ hybridization of *Arc* and *Homer 1a* mRNA and showed strong evidence of functional interplay between HPC and RSC. Acute hippocampal inactivation not only impaired spatial memory, but also altered activity of RSC resulting in loss of behavior-induced *Arc* mRNA expression in this structure [106].

Another behavioral tool used for studying navigation dependent on visual cues is the modification of standard T-maze task, where the front wall of the maze is translucent. One study [107] used this technique to assess whether rats naturally fall into two groups that navigate the maze using either egocentric or allocentric strategy. The proposed criterion of ascribing a strategy to an animal divided the subjects into two groups of similar size, exhibiting different learning dynamics with a statistically significant difference in performance during the early behavioral sessions. Analysis of *Arc* mRNA was conducted to estimate its overall levels in different RSC areas and compare populations activated in each context. Unexpectedly, it was found that the group using egocentric strategy displayed significantly higher activation of both granular and dysgranular rostral RSC, while in the caudal parts of the region the opposite was observed—although without reaching significance. No such dependence was observed in the hippocampus.

### Context processing

Neuroimaging studies demonstrated that RSC is active during processing of contextual information or the formation of contextual association. One of the examples commonly used in rodent studies is contextual fear conditioning, a task that requires association of novel arbitrary visual stimuli to form a representation of a new context. During contextual learning, apart from forming associations between various sensory stimuli that compose the environment, animals must also update those associations when tone and shock are presented later in the acquisition session. Data shows higher levels of FOS and ARC in RSC during both contextual fear learning and the retrieval of contextual fear memories [108, 109]. This effect is NMDA receptor—dependent [110], suggesting that associative learning occurs within this structure. RSC also participates in early post training formation of persistent memory storage. Following FOS level increase in the anterior part of RSC 8–12 h after training, maintenance of contextual fear memory could be established [111]. This increase is associated with persistence of memory. Blocking *Fos* expression by specific antisense oligonucleotides impaired retention 7 days after training without affecting memory 2 days after training [112]. In another study, silencing neural activity in the anterior RSC had a selective impact on event-related memory driven by the auditory conditional stimulus. Inhibition of the posterior RSC selectively impaired memory for the context in which training was conducted. The effect of RSC silencing was indicated by decreased local cellular activity, indexed by lower expression of ZIF268 [113].

As neural representation relies on multimodal stimuli for the best recognition of unmarked places, knowledge from allothetic and idiothetic spatial memory is essential. Building appropriate allothetic or idiothetic frames of reference requires proper recognition and segregation of information derived from relevant and irrelevant stimulus sources. Under the conditions when such strong reference frames are in conflict, involvement of RSC is essential. In the active place avoidance task, RSC damage affected shock sector avoidance only when relevant (room) and irrelevant (arena) cues were available but stayed in conflict [50]. This variant of place avoidance task with relevance of both room and arena stimuli was later used for mapping the *Fos* induction in granular and dysgranular retrosplenial areas. RSC was activated when the process of spatial memory acquisition was in progress, on the first day of training compared with lower level of expression on the third day of training. In granular RSC, the density of FOS nuclei was higher than in the dysgranular part. Moreover, the dysgranular part of RSC appeared not to be essential during long-term memory functioning when rats have already learned effective avoidance, showing lower levels of FOS compared to both control groups [114].

### Consolidation and schema

Recently acquired contextual information is thought to depend primarily on the hippocampus for its successful retrieval. It then time-dependently transforms into enduring stable memories in the process of memory consolidation. According to the prevailing theory, systems consolidation is a process involving the stabilisation of memory traces in the neocortex over time [115, 116]. Expression of immediate early gene *Zif268* in RSC neurons was higher 30 days after task acquisition in a five-arm test, indicating possible involvement of RSC in processing remote spatial memories. Interestingly, the level of FOS protein expression remained high at both: recent and remote time point [117]. In accordance with the theory of memory consolidation, data obtained from rats performing Morris water maze task shows that activity of the RSC during retrieval of spatial memory increased over the course of consolidation, reaching significance between 14 and 30 days. Levels of FOS and ARC proteins increased in correspondence with the age of the memory, however this pattern was not observed with ZIF268 [118]. In another study, *Arc* mRNA levels were elevated following both, recent and remote memory tests [119]. Similarly, increased expression of FOS was observed in the retrosplenial cortex during retrieval of remote contextual memories [97].

While systems consolidation is normally a gradual process, it can occur very rapidly if the basis “schema” into which new information is incorporated has previously been created. In other words, system consolidation in the neocortex can be influenced by what is already known [120]. New traces, new information experienced for only one trial becomes assimilated and rapidly hippocampal-independent in less than 48 h [121]. During assimilation itself, learning within a single trial still requires the hippocampus. IEG activation in the cortex is regulated in part by the relevance of the new information being processed in the hippocampus to an existing cortical schema [122]. Updating schema in the same paired-associate task with two new elements resulted in greater increase of IEG (*Arc* and *Zif268*) expression during encoding in cortical areas such as anterior cingulate cortex, prelimbic cortex, RSC and hippocampal region CA1. In contrast, when rats had to learn a completely new map, increased activation of IEG was limited only to CA1 [123, 124].

### Visual processing

RSC is highly connected with the visual cortex, suggesting that it could be involved in memories which depend on both dorsal and ventral visual pathways and incorporate the “what” component of recognition memory. Such memories allow distinguishing between familiar or novel places and objects being necessary throughout life. Study based on bow-tie shaped maze reports the involvement of the rodent retrosplenial cortex in nonspatial recent memory engagement where rats prefer to explore the least familiar object of a pair. It showed correlation of the number of FOS positive cells in RSC with recency discrimination performance. The recency behavioural tasks are assumed to highlight ‘what/when’ information network [125]. Results reported by de Landeta and others [126] show that the anterior part of RSC is required for the encoding of two of the main recognition features, the “what” and “where” memory components. Functionality of RSC in the non-spatial Y-OR task was specifically required after acquisition, during the consolidation phase, to grant the stabilization of a lasting memory, but not during memory formation itself. Involvement of RSA in recognition memory was confirmed also by the increased level of FOS protein during the consolidation phase [126].

## In vivo IEG imaging

To investigate training-dependent changes in neuronal populations, one needs to be able to track activity in the same region over a period of days. Using genetically-encoded fluorescent markers utilizing immediate early gene promoters allows for observations with cellular resolution and time scale ranging from minutes to hours (Fig. 3C–E). This removes the problem of modifying marker onset and offset times for them to be in line with the true scale of an underlying process, which is present when the markers used are voltage based [127, 128]. Another feature of this approach is that it introduces an onset delay, which can be advantageous in experiment planning. It also allows one to exploit differences in the timelines of activation of different IEGs to investigate neuronal correlates of different steps in a chain of events at the same time [127].

The early 2000s brought the development of transgenic mice where GFP fluorescence was driven by the *Fos* promoter. It was the first time when a fluorescent marker of IEG expression was detected in living cells without signal enhancement [129]. One of the tools that can be used for this purpose is two photon microscopy. This particular technique works especially well for a region such as the retrosplenial cortex due to its location on the top surface of the rodent brain—which means ease of the surgery and no side effects of lesions to be considered [130].

Such an approach, combining the use of this particular strain of mice expressing *FosGfp* fusion gene and time lapse two photon imaging through a cranial window has been utilized to test whether RSC encodes spatial information in a modified Morris water maze design [131]. Basing on the assumption that cells repeatedly expressing *Fos* in subsequent training sessions form a memory trace and the level of fluorescence corresponds directly to the strength of cell activation, this experiment revealed that sessions that required spatial strategy to be employed caused more robust activation of the RSC than non-spatial ones. Moreover, many of those of the RSC layer II neurons that exhibited the strongest difference in activation between spatial and non-spatial tasks form a population that is preferentially reactivated in subsequent sessions, where the mice had to depend on a complex array of allocentric cues.

Two photon in vivo FOS imaging has been also used in an experiment showing that spatial learning in a radial arm maze is accompanied by emergence of context-specific memory engram [132]. This study employed a different type of transgenic animal, *cFos-shGfp* [133]—expressing just the GFP protein under c-fos promoter, in contrast to the *cFos-Gfp* mice, where EGFP-FOS fusion protein is used. This results in significantly shorter half life of the fluorescent protein. Longitudinal imaging revealed that the similarity between the populations of RSC cells activated on consecutive days of training increases with improved performance. Moreover, as the similarity index was also calculated for the final training session and memory retention test around 3 weeks later, this measure, reflecting the stability of the engram, turned out to be predictive of the degree of forgetting. This supports the hypothesis that the retrosplenial cortex is involved in long-term memory formation and retrieval [63, 132].

In a recent study 2-photon in vivo imaging of RSC of two previously mentioned strains of *Fos-Gfp* mice has been used to verify if IEG expression kinetics could be modeled using differential equations for a consecutive, irreversible first-order reaction with a limiting substrate [134]. The temporal profile of fluorescence has been fit to the proposed equation for two types of activation: bicuculline induced seizure and context exposure. Around 75% of identified cells showed significant similarity between experimental data and prediction. Importantly, this has been observed for both types of mice, which suggests that the results might be generalized for further strains, including those based on different IEG promoters. Changing the principle of detection from thresholding the signal at the time point of maximum activation to analysis of expression kinetics also allows for identification of cells responding to two events separated by a given interval. The authors of the paper use this technique to investigate qualities of the RSC ensembles. First, they show that overlap between neuronal populations activated during fear memory retrieval and safe context exposure is at chance level, which might mean that they are unique and independent. Next, they compare the chance of reactivation of a neuron when exposures are separated by different time intervals, finding no temporal linkage, unlike the phenomenon occurring in the hippocampus [135].

Another nuclear IEG, *Zif268*, has also been used in in vivo two photon imaging [136, 137]. The strain of mice that was used was created by the GENSAT project. The animals expressed the EGFP protein under the control of the *Egr1/Zif268* gene promoter. The EGFP fluorescence was monitored in several regions of the neocortex during contextual fear training. It was revealed that in some of those regions, dysgranular retrosplenial cortex among them, there exists a population of layer II neurons that exhibits context-specific response. Moreover, this response was acquired gradually, which suggests that it is not sensory, but memory-related. The identified ensembles continued to produce high levels of activation in the recall trials 3 weeks after the last training session, and those levels presented strong correlation with the amount of freezing of the animal, meaning it is reasonable to conclude that they are involved in long term memory formation [137]. The 2019 study attempted to show that after fear conditioning, extinction and recall trials engage distinct neuronal ensembles in layers II/III of the RSC. It was also suggested that those “safe memory neurons” are a result of hippocampal adult neurogenesis and the integration of those newborn neurons into the circuit [136].

It is important to note that findings based on GENSAT *Egr1-eGfp* mice should be regarded carefully, as a recent study highlights the limitations of this strain, citing lack of colocalization of EGFP and endogenous ZIF268 [138].

## IEG engram manipulation

IEG promoters have been also used to turn on other transgenes that allow not only for identification of IEG-expressing neurons but also for transient manipulation of those ensembles. IEG positive cells can be either silenced or activated by suitable triggers when the expressed transgene encodes light-gated ion channels or ligand-gated G-protein coupled receptors. This is achieved by pairing driver transgene and effector protein, which usually means either using double-transgenic mouse lines or infecting the region of choice in a transgenic animal with a virus [139]. Mouse lines most commonly used for this purpose are those based on the tetracycline transactivator system [133]. In the Tet-Off variant neuronal activation that leads to tetracycline transactivator (driver transgene) expression through IEG promoter is possible only in the time window defined by taking the mice off doxycycline. Neurons that are tagged during this period then express the effector protein. In the simplest form, the effector protein is fluorescent, enabling long term detection of labelled neurons. Combined with native FOS immunolabelling it also allows for the comparison of two neuronal ensembles activated days or weeks apart (Fig. 3D). Using this approach it was possible to reveal that the ensemble activated in RSC during context encoding is similar in size and highly overlapping with the one activated by retrieval at a recent time point [97].

Another version of the Tet-Tag system enables the expression of effector proteins susceptible to an external trigger. Using *fos-tTA* reporter mice allows for long lasting tagging of cells that were active in the duration of the off-dox window. A specific strain, engineered by the Mayford group, expresses tTA, H2B-GFP, and Cre in those cells [133, 140]. This means that said neurons can be identified by their fluorescence. Furthermore, injecting chosen structure with adeno-associated virus encoding double-floxed inverted ArchT gene enables silencing of excitatory neurons of this region after the gene gets inverted and expressed in the presence of Cre. This quality was used in a study exploring the consequences of silencing dCA1 neurons active during fear conditioning. It was revealed that while the total level of *c-Fos* expression in the RSC remains unchanged, a significant drop can be observed in those active during learning—meaning the reactivation of cortical representation is impaired.

Combining FOS tagging with channelrhodopsin expression was used to examine the role and properties of RSC ensembles activated during fear conditioning memory retrieval [141, 142]. Both mentioned studies used *cfos-tTA/tetO-ChEF* bitransgenic mice line. Ensembles tagged during administration of footshocks in context A were compared to those elicited by exposure to the same context and a novel one, showing significantly higher overlap for reentry into context A. Moreover, reactivation of those ensembles by light stimulation in a neutral arena produced a behavioral effect that correlated with initial freezing rate. This shows that RSC takes part in the initial phase of learning, which contributes to the emerging view that cortical structures are engaged not only in the later stages of memory formation and are important for acquisition as well as consolidation. Moreover, this proves that cellular context representations exist in the RSC and they emerge during or shortly after training.

In another experiment the ensemble tagging was conducted in context A one day before fear conditioning either in the same context or in a distinct context B. The light stimulation in a neutral environment revealed that mice shocked in the same context as they were tagged in froze significantly more than those in the second group, meaning that context representation in the RSC is sufficiently stable to produce the association with the aversive stimulus during later training and allow retrieval of this memory [141].

Stimulation of tagged RSC cells in fear conditioned *cfos-tTA/tetO-ChEF* mice was also used to elucidate the influence of ensemble reactivation on the memory. Light was administered both in the awake state [141] and in an offline state, induced by isoflurane anesthesia [142]. In the first case, reactivating the engram associated with the context in which the mice were shocked produced a result similar to fear extinction. For sedated mice, stimulation led to apparent maturation of the memory. Its retrieval became hippocampus independent, much like the case is for remote memories. After ensemble reactivation, mice also displayed context generalization, another sign that consolidation occurred. Results from both of these studies suggest that artificial reactivation of a memory starts a downstream cellular cascade mimicking natural processes, both in respect to behavior and occuring circuit changes.

Another strategy of neuronal ensemble tagging, involving tamoxifen-dependent recombinase CreER^T2^ expressed from the *c-fos* gene locus as a driver for channelrhodopsin expression (TRAP: Targeted Recombination in Active Populations) has been used in a study of RSC role in fear extinction [136, 143]. Administering the 4-OHT shortly after extinction training led to tagging of an ensemble that, upon reactivation, suppressed freezing response during exposure task in a context-specific manner.

In summary, the recently developed techniques based on the expression of IEGs allowed for more systematic analysis of the RSC function. Two main conclusions can be drawn: that a memory trace is being established in this structure from the onset of learning, and that under certain conditions it is capable to support behavior in spatial tasks independently of the hippocampus.

## Summary and conclusions

The detailed summary of all works aimed at mapping immediate early genes in the retrosplenial cortex unveils a complex picture with multiple aspects. In most cases the conclusions overlap and they remain in agreement with data from lesion, electrophysiological and neuroanatomical studies. It is, however, extremely difficult to provide one synthetic explanation of these scattered observations. There have been notable attempts to do so [9, 10, 63, 144], but in each case a substantial number of questions was also raised [144]. Some of these issues might now be addressed after incorporating the latest findings using in vivo IEG mapping and optogenetics.

One of the critical issues regarding the role of RSC in the circuit for spatial memory is its functional relationship with the hippocampal formation and the hippocampus itself [144]. This relationship has been often viewed in the framework of the systems consolidation theory, where hippocampal memory trace is gradually transferred towards new engrams in cortical regions [145] or rapidly incorporated into existing ones [121, 122]. RSC would therefore recapitulate and transform the hippocampal representation (possibly including some unique features), but the final effect would still be a derivative of its initial content.

**Fig. 4:**
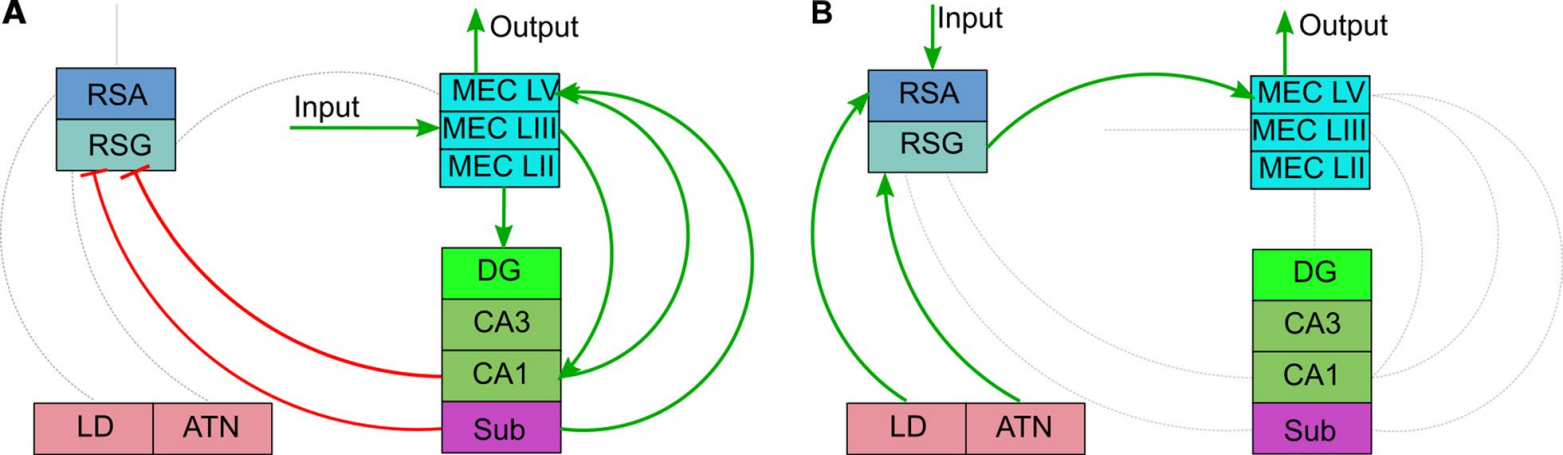
Putative models of “hippocampal” and “retrosplenial” processing of spatial information. **A** Sensory input into superficial layers of MEC is forwarded to the hippocampus (LII MEC to DG and CA3, LIII MEC to CA1). The output of hippocampal processing is fed back to LV MEC and towards cortical areas. RSC is suppressed by inhibitory projections from CA1 and/or subiculum. **B** Sensory input to RSA and RSG is forwarded directly to the output neurons of LV MEC

We propose that the retrosplenial cortex might be capable of establishing its own memory trace, independent of the hippocampal one and parallel to it. Several IEG mapping studies indicate the emergence of such a parallel engram from the onset of learning [110, 112, 117, 119, 126, 131, 132] and their results are confirmed with recent electrophysiological [146, 147] an d behavioral findings [18]. The unique retrosplenial memory trace would be formed on the basis of the direct input from the visual cortex to RSA. Thalamic efferents would also be able to provide information related to directionality, with ATN relaying vestibular input and LD supplementing additional preprocessed visual information. This trace would be supported by other adjacent higher order cortical areas connected directly to RSC (parietal, prefrontal and cingulate cortex). The representation would be more schematic, and based on distinct allothe tic visual landmarks (each encoded individually) rather than one complex, panoramic image of the entire context. It would also include a set of spatial relationships between the landmarks, but not necessarily the impression of context as a whole. As a consequence, only one of the canonical hippocampal functions, pattern separation, could be performed within this circuit. Findings from lesion studies show that the RSC may aid in switching between different categories of cues and facilitate decision making when some of them are in conflict with the others which is in line with the model of retrosplenial cortex as a repository of landmarks and relationships between them. Since spatial memory deficits of lesioned animals are especially prominent during experiments conducted in the dark, proposed importance of visual cues in the RSC mediated navigation is also supported [148]. A similar role has been postulated before [149] and a functional circuit was proposed [11]. Still the retrosplenial cortex was believed to be a part of the hippocampal circuit in these considerations. Interestingly, a possibility of a hippocampus—independent system for context acquisition was suggested and experimentally proven before [150]. From the functional perspective, the effects of RSC processing could be fed directly into the deep layers of MEC [23] and therefore dominate the MEC output, effectively overriding the hippocampal contribution (Fig. 4B). Connectivity [19] and functional data [20, 151] shows that the hippocampus is capable of inhibiting the RSC via direct input from CA1 (Fig. 4A). A more detailed functional study also showed bidirectional modulation of RSC function via direct excitation and feedforward inhibition from the subiculum [18]. The expression of retrosplenial memory would only be possible under conditions where hippocampal inhibition of RSC is suppressed [20]. Under real life circumstances this wo uld be possible when direct input to CA1 from MEC layer III is not supported by the output of the trisynaptic pathway (no hippocampal retrieval). This effect would be particularly important in the case of conflicting outcomes from both circuits, and interaction between RSG and Sub would efficiently control the switch between the two modes of navigation.

**Fig. 5:**
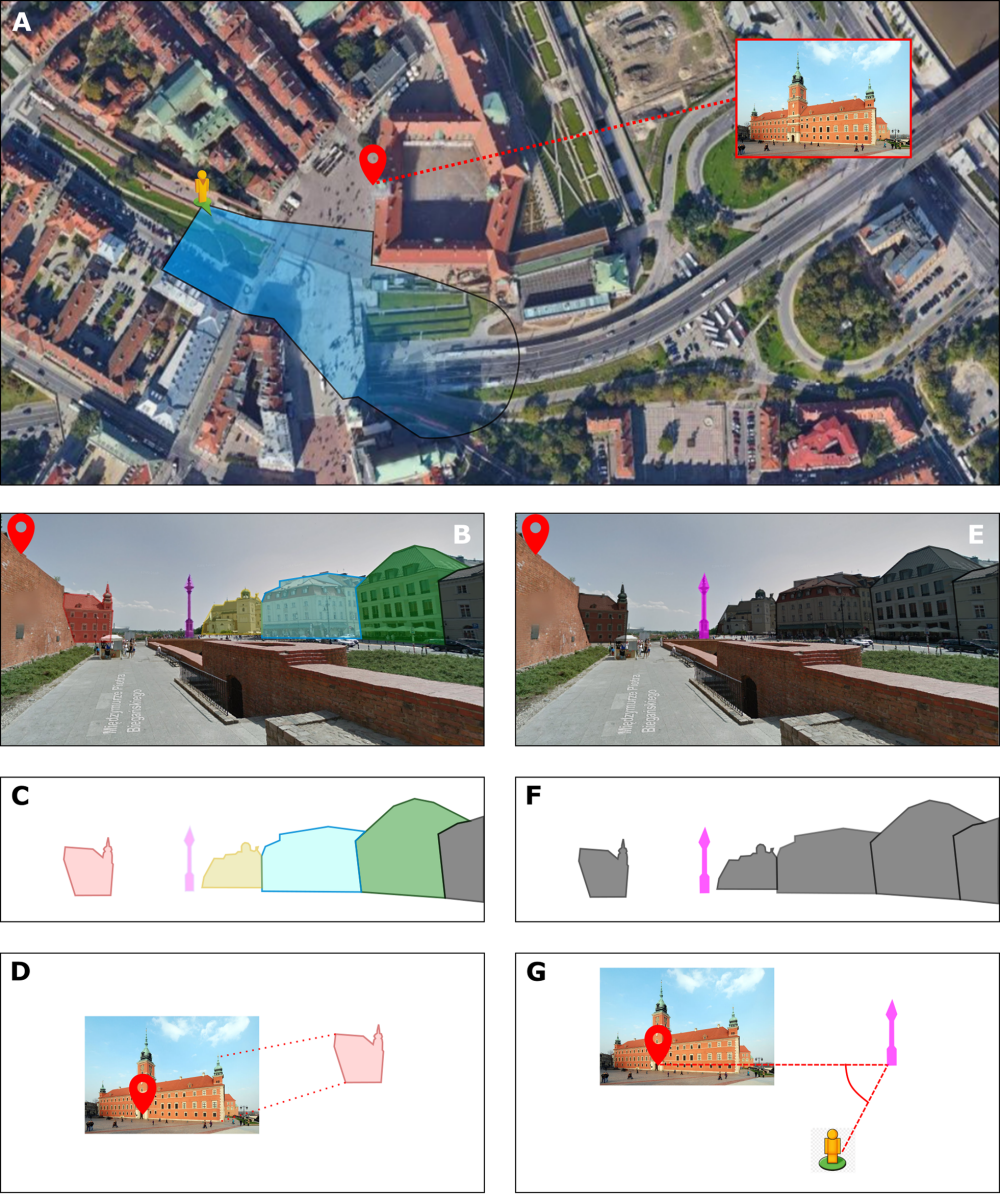
Hypothetical real life example of “hippocampal” and “retrosplenial” mode of navigation. Detailed description provided in the main text. **A** Satellite map of a fragment of Warsaw Old Town, position/direction of the observer and the goal location indicated by icons. Blue polygon indicates visual range of the observer. **B** and **C** individual components of the context encoded by the hippocampus. **D** Detection of goal by pattern completion. **E** and **F** distinctive landmark encoded by the RSC. **G** Detection of goal by relative location. Images credit: Google Maps, Wikipedia

The postulated hypothetical difference between hippocampal and retrosplenial processing of spatial information is illustrated in Fig. 5. A tourist walking along the southern wall of Warsaw Old Town and looking for the Royal Castle would eventually come across the distant view of a fragment of the Castle Square, with the main tower of the castle still obscured by the city walls (Fig. 5A). From this sensory input the hippocampus would instantly acquire a complete “snapshot” consisting of at least four distinguishable buildings on the right side (Fig. 5B, C), a fragment of another building on the left side, and a tall statue (Sigismund’s Column) in the middle of view. Using the same information, but provided by a parallel stream, the retrosplenial cortex would isolate the dominant central landmark and notice a set of spatial relationships between the landmark and the buildings to its sides (Fig. 5E, F). If any prior visual information was available to the tourist (photos from a tourist guide, or recollections from a previous visit), the hippocampus would easily recognize the fragment of the red building to the left as the southern wing of Royal Castle (pattern completion, Fig. 5D). If any map information was also available to the tourist, the retrosplenial cortex would identify the spatial relationship between the Sigismund’s Column and the Royal Castle (left turn at the column, Fig. 5G). In this particular case both systems would lead to successful task completion and the difference between the two navigation modes seems minute. Upon closer examination the hippocampal navigation comes out as much more demanding both in terms of sensory input and processing abilities. Good eyesight and sufficient visibility is needed to form a detailed context representation upon encoding and to retrieve the unique features of the context during retrieval. These demands are less strict in the case of landmark based navigation.

This example shows why the RSC lesions in rodent models often fail to expose spatial memory deficits under experimental conditions. Hippocampal mode of navigation is perfectly capable of supporting spatial tasks under most circumstances. It has been difficult to establish behavioral paradigms in animal models that would allow alternating between the hippocampus and retrosplenial cortex, although several notable attempts have already been successful [50, 148, 152], reviewed in [63]. These reports remain largely in agreement with our model. Studies concerning RSC in object-in-place scene discriminations tasks may further contribute to our interpretation. RSC damage leads to specific impairment in ability to retrieve spatial network of landmarks that has been previously acquired [55]. Fear conditioning on the other hand, the most common behavioral task used in spatial memory research, appears somewhat unsuitable for these experiments as it does not involve the navigational component. The results obtained with this paradigm, although robust, prove difficult to interpret and to advance the model beyond fundamental findings. Assuming the theoretical framework proposed above, it is now necessary to redesign and improve these tasks and to apply them with novel methods of in vivo neuronal activity tracking and controlling. Such an approach would ultimately lead to a more precise description of the functional components of spatial memory circuit. Several predictions could then be formulated and tested experimentally in order to validate our hypothesis. In particular, in an ultimate experiment, using alternative opto/chemogenetic suppression of each circuit during training it should be possible to encode in one animal two separate spatial representations of the same context that are associated with different behavioral outputs. It would then be possible to trigger these behaviors by reinstating each of the engrams. If the retrosplenial trace is indeed dependent on the hippocampus, some redundancy would always remain between the two outputs.

## Data Availability

Not applicable.

## Abbreviations

4-OHT: 4-Hydroxytamoxifen
AMPAR: α-Amino-3-hydroxy-5-methyl-4-isoxazolepropionic acid receptor
AP-1: Activator protein-1 complex
Arc: Activity-regulated cytoskeleton associated protein gene
ArchT: Archaerhodopsin-TP009 gene
ATN: Anterior thalamic nuclei
CA1: Hippocampal (corni ammonu) subregion CA1
CA3: Hippocampal (corni ammonu) subregion CA3
CaM: Calmodulin
catFISH: Cellular compartment analysis of temporal activity by fluorescent in-situ hybridization
c-Fos: C-Fos gene
CNS: Central nervous system
Cre: Cre recombinase
CREB: CAMP response element-binding protein
CreERT2: Cre recombinase fused to a mutant estrogen ligand-binding domain
dCA1: Dorsal part of hippocampal subregion CA1
DG: Dentate gyrus
EGFP: Enhanced green fluorescent protein
EGR-1: Early growth response protein 1
ELK-1: Transcription factor ELK1
FOS: Fos protein
GFP: Green fluorescent protein
H2B-GFP: Human histone H2B and green fluorescent protein fusion
HD: Head direction cells
HPC: Hippocampus
IEGs: Immediate early genes
Jun: Jun gene
JUN: Jun protein
Krox: Krox gene
KROX: Krox protein
Krox-24: Krox-24 gene
LD: Laterodorsal thalamic nucleus
LTP: Long term potentiation
MAPK: Mitogen-activated protein kinases
MB: Mammillary bodies
MEC: Medial entorhinal cortex
MECS: Maximal electroconvulsive shock
mGluR: Metabotropic glutamate receptors
mRNA: Messenger ribonucleic acid
MTT: Mammillothalamic tract
NMDA: N-methyl-d-aspartate
NMDAR: N-methyl-d-aspartate receptor
PaS: Parasubiculum
PoS: Postsubiculum
PrS: Presubiculum
REM: Rapid eye movement
RSA: Agranular retrosplenial cortex
RSC: Retrosplenial cortex
RSG: Granular retrosplenial cortex
RTF: Regulatory transcription factors
Sub: Subiculum
Tet-Off: Variant of inducible tetracycline-controlled transcriptional activation
Tet-Tag: System of inducible tetracycline-controlled transcriptional activation
TRAP: Targeted recombination in active populations
tTA: Tetracycline-controlled transactivator
ZIF268: Zinc finger protein 268
Zif268: Zinc finger protein 268 gene
αCaMKII: Calmodulin-dependent protein kinase IIα
